# Distinctiveness in virological features and pathogenic potentials of subgenotypes D1, D2, D3 and D5 of Hepatitis B virus

**DOI:** 10.1038/s41598-018-26414-4

**Published:** 2018-05-23

**Authors:** Mousumi Khatun, Rajiv Kumar Mondal, Sourina Pal, Ayana Baidya, Debasree Bishnu, Priyanka Banerjee, Amal Kumar Santra, Gopal Krishna Dhali, Soma Banerjee, Abhijit Chowdhury, Simanti Datta

**Affiliations:** 1 Centre for Liver Research, School of Digestive and Liver Diseases, Institute of Post Graduate Medical Education and Research (I.P.G.M.E. & R.), Kolkata, India; 2 Department of Gastroenterology, School of Digestive and Liver Diseases, Institute of Post Graduate Medical Education and Research (I.P.G.M.E. & R.), Kolkata, India; 3 Department of Hepatology, School of Digestive and Liver Diseases, Institute of Post Graduate Medical Education and Research (I.P.G.M.E. & R.), Kolkata, India

## Abstract

Distinct clinical features of HBV infection have been associated with different viral genotype/subgenotype. HBV Genotype-D comprised of 10 subgenotypes, D1–D10, whose clinical implications still remain elusive. We investigated for the first-time, the virologic characteristics and cytopathic effects of four non-recombinant D-subgenotypes, D1/D2/D3/D5. Expressions of viral/host genes were evaluated in Huh7 cells transfected with full-length, linear-monomers of HBV/D-subgenotypes or pGL3-Basic vector carrying subgenotype-specific HBx. Intracellular HBV-DNA and pregenomic-RNA levels were high in D1/D2 than D3/D5. Expressions of PreC-mRNA and HBx were highest for D2 and D1 respectively, whereas PreS2/S-transcript was significantly reduced in D5. Increased apoptotic cell death and marked upregulation in caspase-3/Bax/TNF-R1/FasR/TRAIL-R1/ROS/MCP-1/IP-10/MIP-1β expression were noticed specifically in D2- and also in D3-transfected cells, while D5 resulted in over-expression of ER-stress-markers. D-subgenotype-transfected Huh7 cells were co-cultured with PBMC of healthy-donors or LX-2 cells and significant increase in pro-inflammatory cytokines in PBMC and fibrogenic-markers in LX-2 were noticed in presence of D2/D3. Further, Huh7 cells transfected with D1, in particular and also D5, displayed remarkable induction of EMT-markers and high proliferative/migratory abilities. Collectively, our results demonstrated that D2/D3 were more associated with hepatic apoptosis/inflammation/fibrosis and D1/D5 with increased risk of hepatocarcinogenesis and emphasize the need for determining HBV-subgenotype in clinical practice.

## Introduction

Hepatitis B virus (HBV) is a small, enveloped DNA virus that replicates in human hepatocytes and leads to a wide spectrum of liver diseases, including acute hepatitis, fulminant liver failure, chronic hepatitis, fibrosis, cirrhosis and hepatocellular carcinoma (HCC)^1^. A striking feature of HBV is its remarkable genetic diversity that is mostly attributed to its error-prone replication via reverse transcription as well as to host immune pressure and the genetic processes, such as recombination, insertion/deletion, genetic drift, population dynamics and biogeography^2,3^. Comparisons of HBV sequences from different geographical regions had led to the identification of ten genotypes (A–J), defined by more than 7.5% genome dissimilarity and most genotypes further segregate into subgenotypes that differ from each other by 4–7.5%^3,4^. Currently, genotypes A, B and C have been subdivided in subgenotypes A1–A7, B1–B9 and C1–C16 while 10 subgenotypes of D (D1–D10), 4 subgenotypes of F (F1–F4) and 2 of I (I1–I2) have been described^5^.

It is widely believed that HBV-triggered liver injury is primarily mediated by host immune response to viral proteins, although other studies had suggested that liver pathology may also be caused by endogenous cytopathic effects of the virus itself, even in absence of a functional immune system^6,7^. Among the viral factors, HBV genotype/subgenotype have been implicated in pathogenesis and clinical outcome of HBV-infection^1^. A plethora of country-specific studies had documented that HBV genotype-C is associated with more severe liver disease than genotype-B, whereas genotype-D takes a more aggressive disease course than genotype-A^1,8,9^. However, studies on the clinical relevance of HBV subgenotypes are very limited. High incidence of HCC had been reported in African patients infected with subgenotype-A1, in Asian patients carrying B2–B5 and C2 and in Alaskan natives with subgenotype-F1^10–12^. In contrast, A2, B1 and B6-infected individuals tend to run a more indolent disease course^1^. Although HBV/D is the most widespread of all HBV genotypes, the impacts of the various sub-genotypes of HBV/D on disease progression have not been adequately explored. Of the ten D-subgenotypes so far identified, D1–D7 and D10 are non-recombinant types while D8 and D9 are D/E and D/C recombinants respectively^3,5^. Six out of the ten D-subgenotypes, namely D1–D5 and D9 had been reported from different parts of India^3,13,14^. In the present study, we performed a comprehensive analysis of the virological features and cytopathic effects of four non-recombinant D-subgenotypes, D1, D2, D3 and D5, prevalent in Eastern India to gain an insight into their potential contribution to disease progression, which in turn will help in the design of appropriate surveillance and therapeutic strategies for the management of HBV/D-infected patients.

## Results

### HBV isolates/clones used for *in*-vitro assays

To study the distinct virologic characteristics and pathogenic potentials of four non-recombinant HBV/D-subgenotypes (D1/D2/D3/D5), we cloned their full-length genomes in pJET1.2/blunt vector and selected a particular clone of a specific D-subgenotype whose genome was devoid of mutations, such as A_1762_T/G_1764_A in basal core promoter (BCP), G_1896_A in Pre-core region or any significant mutation in viral polymerase/surface antigen (HBsAg) that has been reported to be associated with primary drug resistance, interference with HBV replication and HBsAg expression as well as any other insertions/deletions^15^. The nucleotide sequences of subgenotypes D1, D2, D3 and D5 used for functional studies are available in the GenBank database (http://www.ncbi.nlm.nih.gov/GenBank/index.html) under accession numbers KM524342, KM524351, KU668446 and GQ205378 respectively.

### Expression of viral transcripts among different HBV/D-subgenotypes

**Figure 1:**
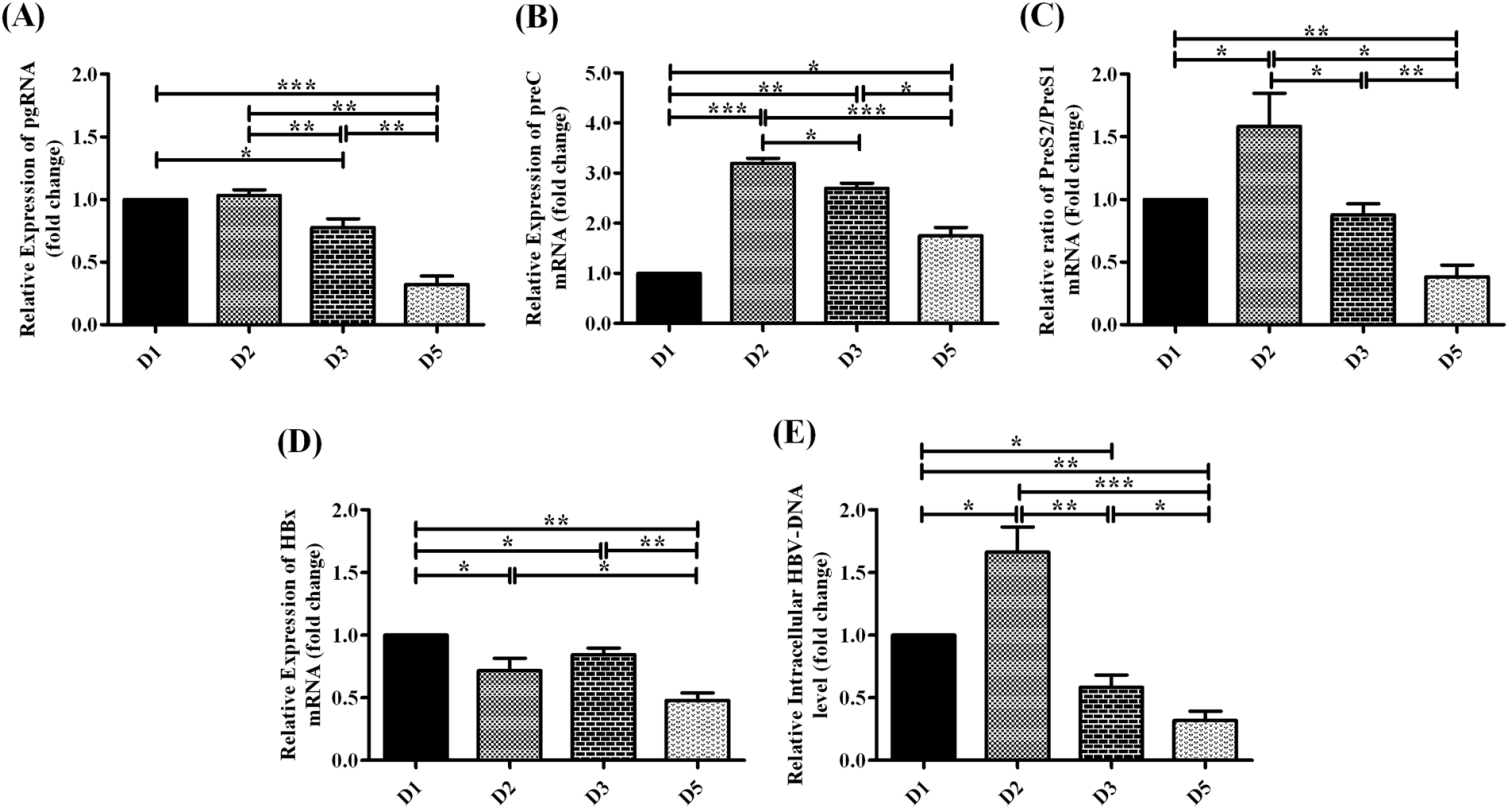
Expression of HBV-mRNAs and intracellular core-associated HBV-DNA measured by real-time PCR following the transfection of full-length linear monomers of specific HBV/D-subgenotype, D1, D2, D3 and D5 into Huh7 cells. Relative expression of **(A)** pregenomic-RNA (pgRNA), **(B)** preC-mRNA, **(C)** ratio of PreS2/PreS1 mRNA, **(D)** HBx mRNA, **(E)** level of intracellular core-associated HBV-DNA among different HBV/D-subgenotypes. Paired t-test p values; *p < 0.05, **p < 0.01, ***p < 0.001.

The pregenomic-RNA (pgRNA) (3.5 kb) is the major HBV transcript that serves as a template for viral replication via reverse transcription as well as for translation of polymerase and core proteins^15^. We quantified and determined the relative abundance of pgRNA in Huh7 cells transfected with HBV/D1, D2, D3 and D5 by real-time PCR. In transfected Huh7 cells, HBV/D2 and D1 directed a much higher level of pgRNA than D3 and D5, with D5 showing the lowest transcript level (Fig. 1A).

The PreC-mRNA is utilized for the synthesis of Pre-core protein, a precursor of hepatitis B e-antigen (HBeAg)^16^ and its expression was highest for HBV/D2 followed by D3, D5 and D1 (Fig. 1B).

The largest subgenomic-mRNA of HBV is 2.4 kb preS1-mRNA that yields the large surface protein (L) while the shorter preS2/S transcript (2.1 kb) gives rise to the middle (M) and small surface proteins (S/HBsAg)^15^. Reduced levels of preS2/S-mRNA, resulting in low preS2/S- to PreS1-mRNA ratio was observed for D5, implying diminished HBsAg expression (Fig. 1C). However, HBV/D2 over-expressed preS2/S transcript and exhibited the highest preS2/S- to PreS1-mRNA ratio, which was followed by D1 and D3, where the ratios were comparable.

The smallest HBx gene overlaps with other HBV-ORFs^17^ making it difficult to evaluate its expression by real-time PCR in Huh7 cells transfected with full-length HBV genome. To overcome this, we cloned the complete HBx gene from each HBV/D subgenotype together with its native promoter region into pGL3 Basic vector and used the pGL3-HBx construct for transfection, such that the transcription of HBx would be regulated by its own promoter. D1-specific and D5-specific HBx showed the highest and lowest level of expression respectively in Huh7 cells, whereas D2- and D3-derived HBx were expressed at almost equivalent levels (Fig. 1D).

To ascertain that the variation in the expression pattern of different viral genes was not due to transfection related alterations, we measured 18 S rRNA expression by real-time PCR in different HBV/D-subgenotype and pGL3-Basic vector-transfected as well as untransfected Huh7 cells. The 18S rRNA expression was found to be similar in all transfected/untransfected Huh7 cells (Supplementary Figure S1).

### Replication efficiency of HBV/D-subgenotypes

Replication efficiency of different D-subgenotype was determined by quantifying the intracellular core-associated HBV-DNA in transfected Huh7 cells. Marked variation in HBV-DNA level was noted across subgenotypes with HBV/D2 displaying the highest level followed by D1 and D3, while the lowest DNA level was observed for D5 (Fig. 1E).

HBV genotype D is characterized by higher rate towards HBeAg-negative chronic infection^2^ and mutations in BCP (A1762T/G1764A) and in Pre-core gene (G1896A) that reduce and abolish the production of HBeAg respectively^15^, are commonly observed in the genome of these viral isolates. BCP mutations are known to impact the replication capacity of the virus^18^ and we investigated the effect of these mutations on the replication efficiency of various D-subgenotypes by introducing the double 1762T and 1764A mutations in the backbone of wild-type HBV/D1, D2, D3 and D5 by site-directed mutagenesis and transfecting these constructs in Huh 7 cells along with their wild-type counterparts. Regardless of HBV D-subgenotype, HBV genomes carrying the BCP mutations exhibited an enhanced replication phenotype than their corresponding wild-type (Supplementary Figure S2). However, the overall pattern of replication of these different BCP-containing D-subgenotypes closely resembles the trend depicted by their parental wild-type strains.

### Apoptosis-inducing abilities of HBV/D-subgenotypes

**Figure 2:**
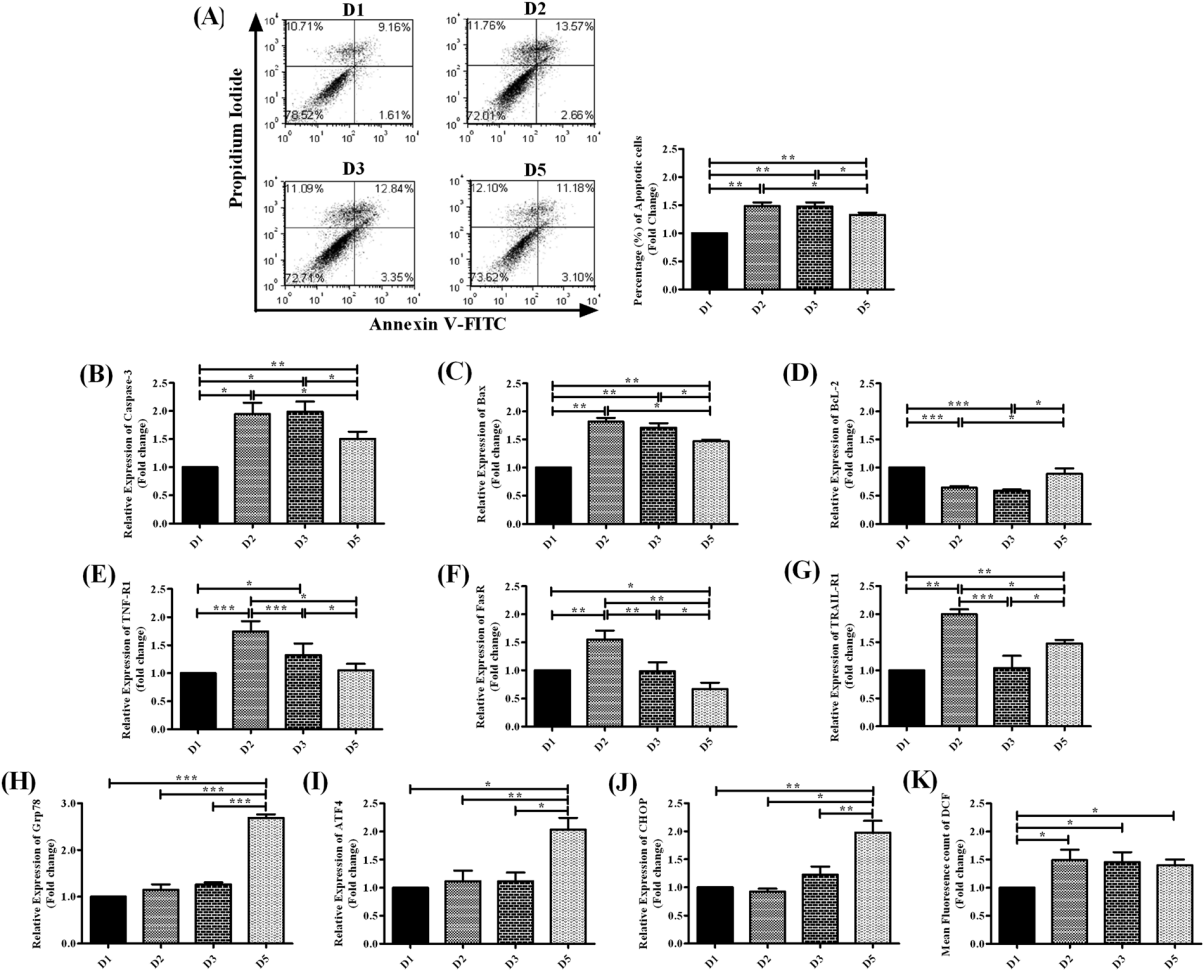
Evaluation of apoptosis and assessment of variety of genes and molecular events associated with intrinsic and extrinsic pathways of apoptosis in Huh7 cells transfected with full-length linear monomers of HBV/D1, D2, D3 and D5. Apoptotic cell death was determined by Annexin-V-FITC and propidium idodide (PI) staining method using flow cytometry. **(A)** A representative experiment showing percentage of apoptotic cells. In each panel the lower left quadrant shows both Annexin-V and PI negative cells (live cells), lower right quadrant shows only Annexin-V positive cells (early apoptotic cells), upper right quadrant shows both Annexin-V and PI positive cells (Late apoptotic cells), upper left quadrant shows only PI positive cells (necrotic cells). The sum of the percentages of both early and late apoptotic cells in presence of different D-subgenotype was determined and the data was represented in mean ± SD bar (n = 3). Relative expression of **(B)** caspase-3, **(C)** Bax, **(D)** Bcl-2, **(E)** TNF-R1, **(F)** FasR, **(G)** TRAIL-R1, **(H)** Grp78, **(I)** ATF4 and **(J)** CHOP were evaluated by real-time PCR in Huh7 cells transfected with D-subgenotype. The levels of **(K)** ROS in D-subgenotype transfected Huh7 cells were determined by DCFDA assay. Paired t-test p values; *p < 0.05, **p < 0.01, ***p < 0.001.

Apoptosis is an important mediator of liver disease caused by HBV infection^19,20^. We investigated the potential of diverse D-subgenotypes to induce apoptosis by Annexin-V/PI assay. The cells positive for Annexin-V and negative for PI staining are considered to be undergoing early stages of apoptosis while cells that stain intensely with both Annexin-V and PI are considered to be in late apoptosis^21^. As shown in Fig. 2A in a representative experiment, the overall fractions of Annexin-positive Huh 7 cells (i.e. sum of Annexin V^+^PI^−^ and Annexin V^+^PI^+^ cells) were 16.23%, 16.19%, 14.28% and 10.77% in presence of D2, D3, D5 and D1 respectively. Overall, significantly enhanced cell death was observed in D2- and D3-transfected Huh7 cells than D5 and D1 and the apoptotic events were least pronounced in cells harbouring D1 (Fig. 2A).

We further interrogated the mechanisms of cell death induced by different D-subgenotypes by studying the alterations in expression of a variety of genes associated with extrinsic- and intrinsic-pathways of apoptosis and evaluating ROS production in transfected Huh7 cells.

#### Expression of caspase-3, Bcl-2 and Bax

Cysteine-aspartic proteases 3 (Caspase-3) is the crucial executioner caspase whose activation is the key event in both extrinsic- and intrinsic-pathways^22^. In congruence with high rate of apoptosis seen in D2- and D3-transfected Huh7 cells, we perceived a significant upregulation in caspase-3 expression in these two subgenotypes, as compared to D5 and D1. D1-transfected cells exhibited the lowest level of caspase-3 (Fig. 2B).

BCL-2-family proteins regulate and mediate the apoptosis and its members comprised of pro-apoptotic BCL2 associated X protein (BAX) and anti-apoptotic B-cell lymphoma 2 (BCL-2) proteins^19^. The expression of Bax was found to be high in Huh7 cells transfected with D2 and D3 but was significantly diminished in presence of D5 particularly D1. Inversely, Bcl-2 level was significantly upregulated in D1 as well as D5-transfected cells, while it is downregulated in D2 and D3 (Fig. 2C,D).

#### Expression of death receptors (DR)

Extrinsic apoptosis is triggered by the engagement of DRs, such as, Tumor Necrosis Factor Receptor 1 (TNF-R1), FAS receptor (FasR/CD95) and TNF-related apoptosis-inducing ligand receptor 1 (TRAIL-R1) by their cognate ligands that eventually lead to the activation of caspases^22^. To evaluate if DR expression is altered by different D-subgenotypes, we transfected Huh7 with HBV/D1, D2, D3 and D5 and examined the expression of DRs by real-time PCR. A significantly high expression of TNF-R1 was noticed in D2-transfected cells, followed by D3 while its expression was low in case of D1 and D5. However, the highest level of FasR expression was perceived in Huh7 cells transfected with D2, which was followed by D3 and D1, whereas D5 exhibited the lowest level of FasR. With regard to TRAIL-R1, the expression pattern was different in that the presence of D2, principally, as well as D5 also upregulated TRAIL-R1 (Fig. 2E–G).

#### Expression of endoplasmic reticulum (ER)-stress-related genes

It is well recognised that over-expression/accumulation of viral proteins often induces ER-stress and initiate a series of signal transduction cascades, known as unfolded protein response (UPR)^23,24^. The key components of UPR include 78 kDa glucose-regulated protein (Grp78), Activating transcription factor 4 (ATF4) and C/EBP homologous protein (CHOP) and apoptotic cell death ensues by ATF4-CHOP-mediated induction of pro-apoptotic genes and suppression of anti-apoptotic Bcl-2 protein synthesis^22^.

We examined the expression of ER-stress markers in Huh7 cells transfected with D-subgenotypes. While the presence of D1, D2 and D3 induced similar levels of Grp78, ATF4 and CHOP, D5 resulted in significant augmentation in expression of these markers, implying a greater ability of D5 to potentiate ER-stress (Fig. 2H–J).

#### Generation of ROS

ROS play an important role in apoptosis induction^19,25^. To assess the effects of D-subgenotypes on induction of ROS, we determined the production of ROS in transfected Huh7 cells using 2,7-dichlorofluorescein-diacetate (DCF-DA) probe, which can be converted to a green fluorescent product dichlorofluorescein (DCF) and as evident from increasing DCF fluorescence, D2-, D3- and D5-transfected Huh7 cells exhibited enhanced rate of oxidative ROS formation, while HBV/D1 showed the least capacity to increase ROS level (Fig. 2K).

### Abilities of HBV/D-subgenotypes in inducing inflammatory response

HBV infection is characterized by a sustained hepatic inflammation in which chemokines and chemokine-receptors orchestrate the recruitment of inflammatory cells to the liver^26^.

#### Expression of chemokines

**Figure 3:**
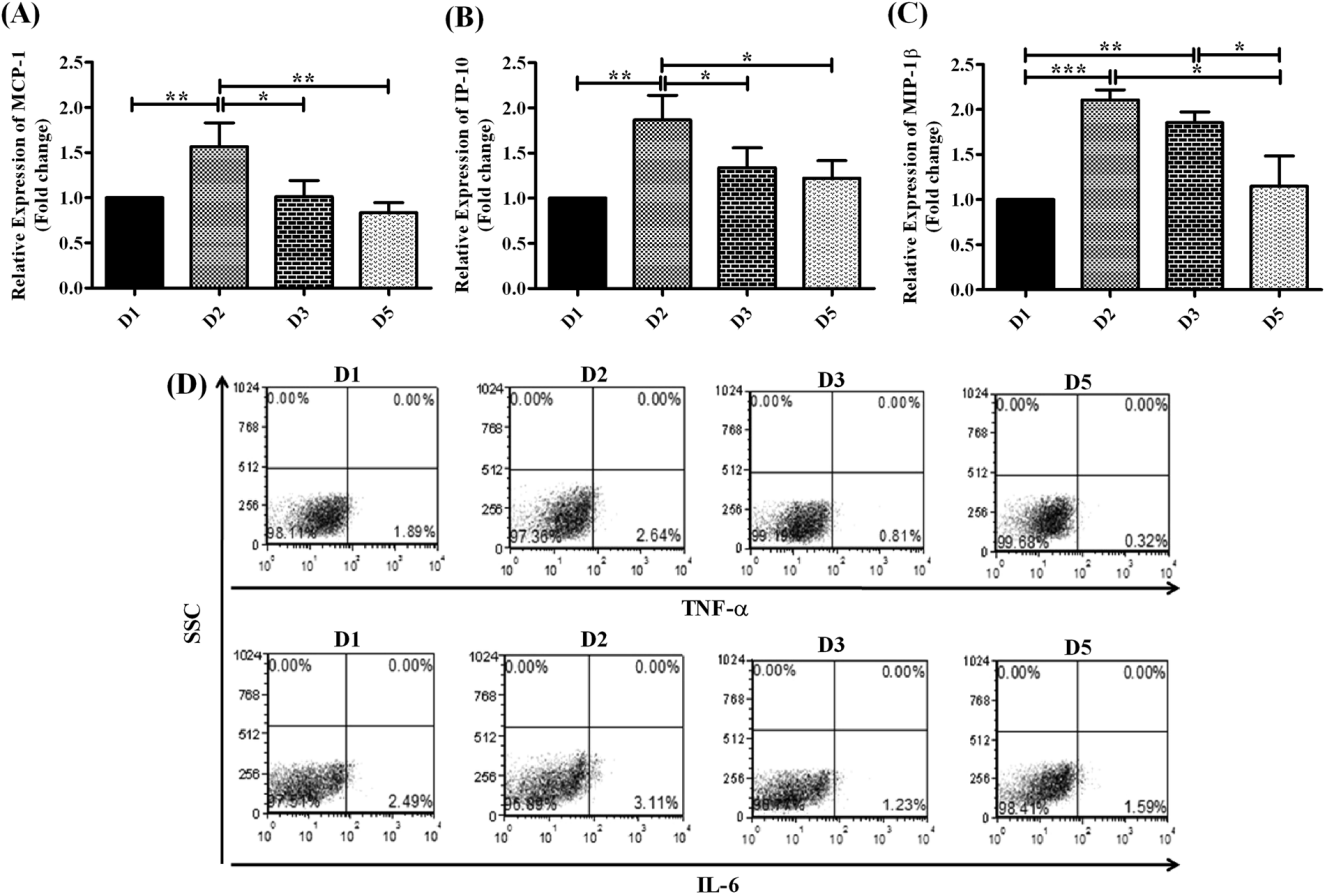
Inflammatory response induced by different HBV/D-subgenotype. Relative expression of chemokines **(A)** MCP-1, **(B)** IP-10 and **(C)** MIP-1β as determined by real-time PCR in Huh7 cells transfected with full-length, linear monomers of different HBV/D-subgenotype. **(D)** A representative diagram depicting frequencies of TNF-α and IL-6 expressing PBMC as evaluated by Flow cytometry following co-culture of Huh7 cells transfected with D1, D2, D3 and D5 with PBMC from healthy donor. Paired t-test p values; *p < 0.05, **p < 0.01, ***p < 0.001.

We investigated the variations in expression of CC-chemokines, macrophage inflammatory protein 1β (MIP-1β) and monocyte chemoattractant protein-1 (MCP-1) that are chemotactic for monocytes and T-cells and CXC-chemokine, interferon-gamma inducible protein 10 (IP-10) that directs T-cell chemotaxis^26–28^ in Huh7 cells transfected with HBV/D1, D2, D3 and D5. It was observed that the expression of both MCP-1 and IP-10 was most profound in D2-transfected cells than in other D-subgenotypes. On the other hand, the level of MIP-1β was comparable in cells harbouring D2 and D3 but was significantly low in D5 and D1 (Fig. 3A–C).

#### Expression of pro-inflammatory cytokines by PBMC

The pattern of cytokines secreted by immune cells at the site of viral replication could influence the outcome of infection^26,29,30^. We evaluated the impact of different D-subgenotypes on PBMC cytokine expression by co-culturing PBMC of healthy donors with Huh7 cells transfected with D-subgenotypes. Exposure to D2-transfected cells resulted in increased production of pro-inflammatory cytokines, Interleukin 6 (IL-6) and Tumor Necrosis Factor α (TNF-α) in PMBC, indicating an enhanced propensity of D2 to elicit a strong inflammatory response than others. However, D1 also showed a high ability to activate the immune cells to secrete these two cytokines (Fig. 3D).

### Potentials of HBV/D-subgenotypes in inducing hepatic fibrosis

Cell death and inflammation constitute two characteristic and intricately-linked features of chronic liver disease that promote the development of fibrosis. The main executioner of fibrosis is hepatic stellate cells (HSC) that receive a wide range of signals from infected/injured hepatocytes and the perturbed hepatic microenvironment, most of these being mediated by cytokines^31^.

#### Expression of CTGF

**Figure 4:**
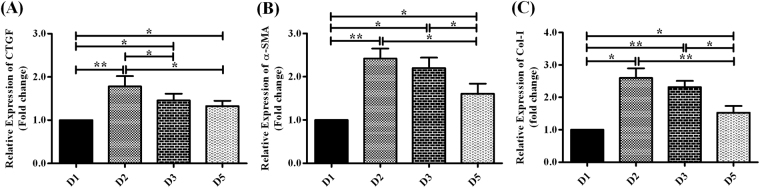
Ability of HBV/D-subgenotypes in inducing fibrogenesis. Relative expression of **(A)** CTGF was determined by real-time PCR in Huh7 cells transfected with HBV/D-subgenotype. Relative expression of **(B)** α-SMA and **(C)** Col-I were assessed in LX-2 cells following co-culture with Huh7 cells transfected with D1, D2, D3 and D5. Paired t-test p values; *p < 0.05, **p < 0.01.

Connective tissue growth factor (CTGF) has been shown to play a key role in the development of hepatic fibrosis^31,32^ and we assessed its expression in Huh7 cells transfected with different HBV/D-subgenotype by real-time PCR. The most prominent CTGF expression was noticed in D2-transfected cells, followed by D3 and D5, whereas CTGF expression was lowest in presence of D1 (Fig. 4A).

#### Expression of α-SMA and Col-I by LX-2 cells

We investigated the aptitude of different HBV/D-subgenotypes in promoting HSC activation and function by co-culturing HBV-transfected Huh7 with LX-2 cells in transwell plates and determining the expression of α-SMA and Col-I in LX-2 cells by real time PCR. Both α-SMA and Col-I-mRNA levels were found to be significantly enhanced in LX-2 cells in presence of HBV/D2 and D3 but were significantly low in D5- and particularly in D1-exposed LX-2 cells (Fig. 4B,C).

### Abilities of HBV/D-subgenotypes to induce tumorigenesis

Chronic HBV infection (CHI) has been linked epidemiologically to the development of HCC^1^ and we explored the contribution of HBV/D-subgenotypes in tumorigenesis.

#### Expression of markers of Epithelial-mesenchymal transition (EMT)

**Figure 5:**
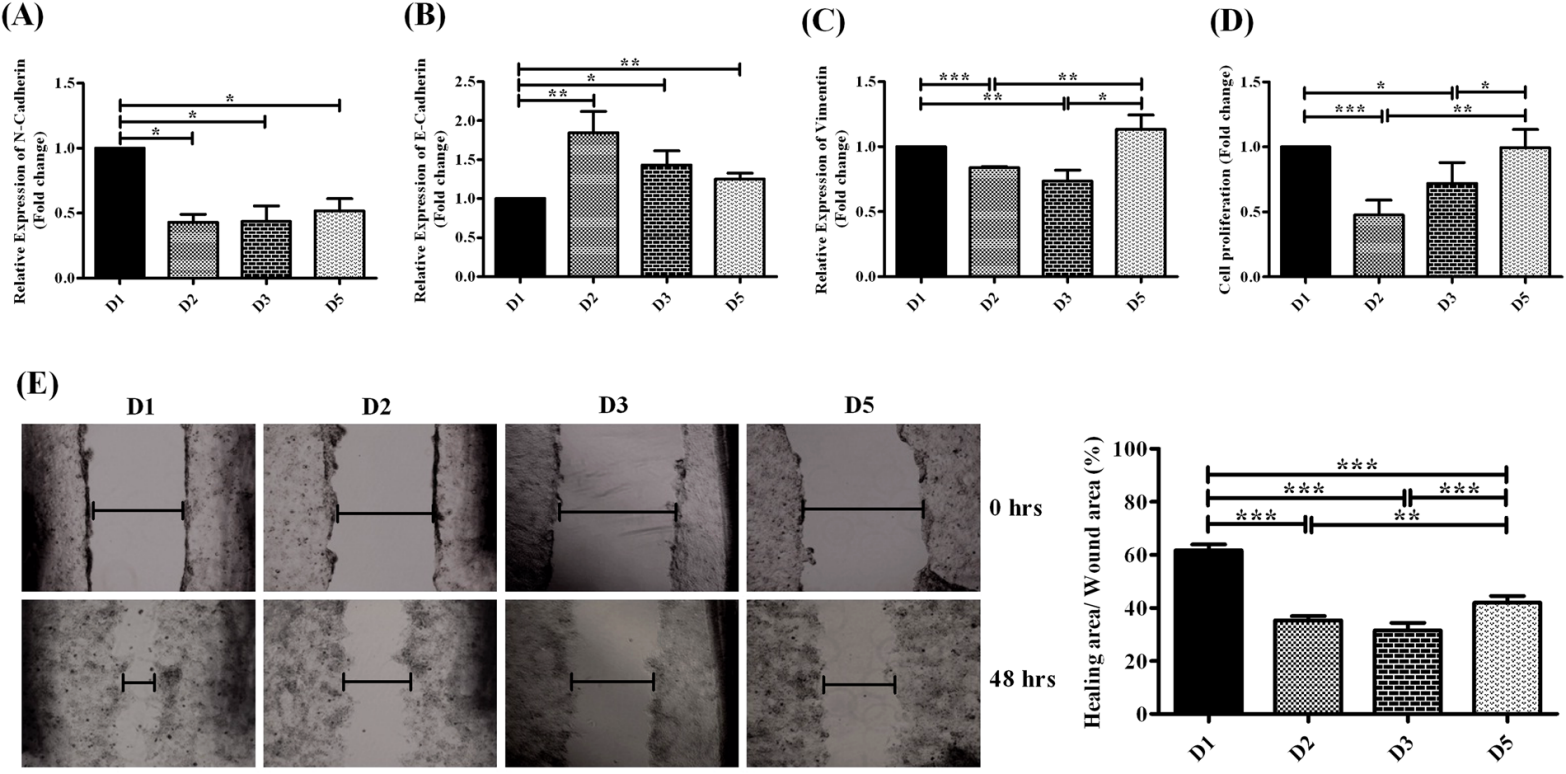
Efficacy of different HBV/D-subgenotype in induction of tumorigenesis. Relative expression of **(A)** N-cadherin, **(B)** E-cadherin and **(C)** Vimentin as determined by real-time PCR in D1, D2, D3 and D5 transfected Huh7 cells. **(D)** Cellular proliferation capabilities were measured by MTT assay in different HBV/D-subgenotype transfected Huh7 cells. **(E)** Cellular migration of transfected Huh7 cells was evaluated by wound healing assay. Representative field (x100) shows wound healing for D1, D2, D3 and D5 transfected Huh7 cells at 48 hrs of scratching. The graph shows the percentage of wound healing area determined by ImageJ software (mean ± SD, n = 3). Paired t-test p values; *p < 0.05, **p < 0.01, ***p < 0.001.

The transdifferentiation of epithelial cells into motile mesenchymal cells, commonly known as EMT, plays a pivotal role in tumor progression/metastasis and is typified by downregulation of E-cadherin and over-expression of N-cadherin and vimentin^33^. We studied the potentiality of different HBV D-subgenotype in inducing EMT in hepatocytes by examining the expression levels of different EMT markers following their transfection in Huh7 cells and detected a significant increase in N-cadherin expression along with a concomitant decline in E-cadherin level in D1-transfected Huh7 cells relative to other D-subgenotypes (Fig. 5A,B). However, substantial elevation in vimentin-mRNA levels were observed in Huh7 cells in presence of both D1 and D5, as compared to D2 and D3 (Fig. 5C), suggesting D1, in particular and also D5 could promote EMT.

#### Proliferation abilities of D-subgenotypes transfected Huh7 cells

The ability of different HBV/D-subgenotype to induce cellular proliferation was evaluated by 3-(4,5-dimethylthiazol-2-yl)-2,5-diphenyltetrazolium bromide (MTT) assay. Huh 7 cells transfected with D1 and D5 featured significantly increased cell proliferation relative to D2 and D3 (Fig. 5D).

#### Migration capabilities of D-subgenotype transfected Huh7 cells

The wound-healing assay was performed to examine the migration ability of Huh7 cells transfected with different D-subgenotype. The results demonstrated that the highest migratory activity was exhibited by cells transfected with HBV/D1, which was closely followed by D5-transfected cells, while cells carrying D2 and D3 showed a much slower healing of wound (Fig. 5E).

## Discussion

Using *in vitro* transfection system in Huh7 cells, the present study has for the first time identified distinct virological differences among four non-recombinant D-subgenotypes, D1, D2, D3 and D5, uncovered their endogenous effects in inducing apoptosis, inflammation, fibrosis and tumorigenesis, the key clinical events associated with HBV infection and thereby established the abilities of these different D-subgenotypes in evoking unique patterns of disease progression and reinforce the importance of screening for HBV subgenotype in infected patients.

Major differences in replicative capacity and protein expression were identified across the D-subgenotypes which may be partly attributed to subgenotype-specific unique signature residues in viral polymerase^34^ and to variations in sequences of the regulatory elements in their genomes. Higher replication was generally observed for subgenotypes D2 and D1 and lower replication for D3 and D5. HBV replication generally mirrored the level of pgRNA, with D2 exhibiting the highest and D5, the lowest levels of core-associated HBV-DNA as well as pgRNA. Although equivalent levels of pgRNA were detected in D1 and D2, yet HBV-DNA in D1 was found to be less than D2, implying difference in activities of subgenotype-specific viral polymerase. Assuming that the main findings in Huh7 cells can be extrapolated to in vivo HBV infection, the variations in overall replication capacity of D-subgenotypes may underlie some of the key differences in clinical features associated with these subgenotypes. It had been reported that HBV/D2 was the major circulating D-subgenotype in patients with acute HBV infection in eastern India^35^ as well as in Japan^36^ and its enhanced replication capacity correlated with high viremia titers and elevated ALT levels in these patients. In contrast, D5 was conspicuously absent in the acute hepatitis B patients^35^. However, among the chronically HBV/D infected patients, the viral load had been found to be highest in patients carrying HBV/D1^37^. On the other hand, occult HBV isolates derived from HBsAg-negative carriers with very low HBV-DNA, mostly belonged to subgenotype-D3 and D5^37^. The abundance of PreC-mRNA coding for HBeAg precursor protein, also varied across the D-subgenotypes. HBeAg has often been used clinically as an index of viral replication^38^ and we observed that the expression pattern of PreC-mRNA also closely reflected the levels of intracellular core-associated HBV-DNA in cases of D2, D3 and D5. However, HBV/D1 exhibited the lowest PreC-mRNA despite its high replication. It had been suggested that reduction in HBeAg in parallel with the enhancement of viral DNA production correlate with an exacerbation of liver injury^39^. Indeed, in an outbreak of HBV with high mortality in Western India, HBV/D1 was found to be associated with fulminant hepatic failure while D2 was most prevalent in cases of self-limiting acute viral hepatitis^40^. Genotype D has been particularly found to be associated with HBeAg-negative chronic hepatitis B (CHB) and BCP mutations had been frequently encountered in the viral species in these CHB patients^2^. Interestingly, the prevalence of BCP mutations had been found to be specifically high in patients infected with HBV/D5^37^. We noted that the presence of BCP mutations increase the replication capacity of all the D-subgenotypes when compared with their wild-type counterparts, suggesting that D5-infected HBeAg-negative patients should be closely monitored for HBV-DNA levels.

It is widely believed that alteration in proper stoichiometry between L- and S-envelope proteins of HBV not only hinder the secretion of HBsAg and virions from the infected hepatocytes but also can cause ER-stress owing to intracellular retention of envelope proteins^24,41^. A markedly reduced synthesis of preS2/S-mRNA was perceived in Huh7 cells transfected with HBV/D5, indicating that D5, despite being a poor replicator, could specifically activate ER-stress by driving an aberrant S/L-protein ratio and thus may contribute to disease progression via ER-stress dependant pathways.

Of the different HBV proteins, HBx is a promiscuous transactivator of viral and cellular promoters and is known to play an important role in HCC development^19,22,42^. HBx expression was found to be particularly high in subgenotype-D1, thereby advocating a predilection of D1 to promote HCC.

HBV infection is known to cause an inflammatory reaction in liver accompanied by hepatocyte death, activation of HSCs and compensatory liver cell regeneration and repeated cycles of these events often lead to increased incidence of fibrosis, cirrhosis and HCC^19,22^. Hepadnavirus-induced apoptotic cell death and cytopathic effects have been described in several experimental model systems^43^. A previous study reported that out of HBV-genotypes A, B and C, HBV/B led to higher apoptosis of HepG2 cells^44^. Here we demonstrated that HBV/D2, in particular, and to lesser extent, D3 and D5 could incite apoptosis of Huh7 cells. Based on the expression profile of markers of intrinsic/extrinsic apoptotic pathways, it has become increasingly apparent that D2 and D3 exert the apoptotic function by activating mitochondrion-mediated as well as DR-dependent pathways, whereas HBV/D5 potentiate cell death primarily via ER-stress. The high TRAIL-R1 expression seen in Huh7 cells carrying D5 appeared to be mediated through induction of CHOP^45^. Conversely, HBV/D1 confers relative resistance to apoptosis, which could be partly explained by D1-driven over-expression of HBx that had been shown to inhibit apoptosis^19,46,47^, although the pro-apoptotic function of HBx had also been described in some studies^20,48^.

Chemokine-directed immune cell infiltration to the liver drives the development of chronic inflammation during HBV infection. In HBV transgenic mouse model, neutralization of chemokines had been shown to reduce lymphomononuclear cell recruitment and consequently the severity of liver disease^49^. We noted robust production of chemokines in D2-transfected Huh7 cells and to a lesser extent in presence of D3, signifying the potential of these subgenotypes to promote the migration of immune cells to the liver lobule, thereby resulting in amplification of intrahepatic inflammation and hepatocyte damage. There is abundant evidence that inflammation involves type-1 cytokine-signalling through IL-1β, TNFα and IL-6^26^. Commensurate with D2-mediated heightened chemokine expression, an increased production of cytokines, TNFα and IL-6 by mononuclear cells was also noticed following co-culture of D2-transfected Huh7 cells and PBMC, which further highlighted the inflammation-inducing capacity of HBV/D2. Interestingly, D1 also triggered a modest level of cytokine production from PBMC and it seems plausible that the high replication rate of both D2 and D1 and consequently high antigen concentration could have stimulated efficient cytokine production by immune cells. Moreover, HBV/D2 also displayed superior aptitude in inducing HSC activation, followed by D3, which could be partly attributed to their abilities in driving the augmented release of fibrogenic mediators, like CTGF from hepatocytes or in causing enhanced hepatocyte apoptosis. Apoptotic cells release ATP or UTP into the extracellular milieu that are involved in heightened purinergic P2Y receptor activation in HSC and impacts procollagen-I transcription^31,50,51^. Additionally, denatured DNA from apoptotic hepatocytes had been shown to induce the differentiation of HSC via Toll-like receptor-9 (TLR-9) expressed in HSCs^52^. HBV/D1 and D5, on the other hand exerted less stimulatory effect on HSC.

Given the overwhelming evidence for a causal role of HBV in HCC development, we attempted to assess the differential impact of different D-subgenotype in hepatocarcinogenesis. Our results indicated that HBV/D1, in particular, as well as D5 were associated with higher risks of HCC. The high oncogenic potential of D1 could be linked to the high expression of HBx that has been implicated in mediating the alterations in DNA methylation, histone modifications and miRNA expression in host cells, thereby contributing to malignant transformation^53^. The downregulation of FasR, DRs and pro-apoptotic proteins in D1-transfected cells and consequently their reduced sensitivity to apoptosis and high proliferative ability could further trigger the development of liver cancer. However, a high expression of mesenchymal-marker, Vimentin and acquisition of increased cellular proliferation and migratory capabilities was also observed in D5-transfected Huh7 cells. Recent studies had depicted that ER-stress may activate an EMT-like state^54^ and induction of tumorigenesis by D5 correlates with increasing ER-stress caused by D5. In addition, chronic apoptotic stimulus, high ROS generation and inflammatory environment could also predispose to cancer development^19,55^, implying that long-term carriage of D2 and D3 may also result in malignancy in the background of advanced fibrosis and cirrhosis.

Thus taken together, the study unravelled the previously unrecognized differences in virological properties and pathogenic potential of different subgenotypes of HBV/D. Although D2 and D1 showed high replication rate, D2 would provoke hepatocyte apoptosis and robust production of chemokines and inflammatory cytokines, expand inflammation and induce the fibrotic processes that might culminate to cirrhosis or even HCC. In contrast, D1 would restrict apoptosis and directly promote the development of HCC, often bypassing the fibrosis step. The slow replicating subgenotypes, D3 and D5, on the other hand, showed a close resemblance to D2 and D1 respectively, in terms of their biological properties. However, a limitation of this study is that we included only one clone per D-subgenotype for functional studies. Nonetheless the study highlighted that determination of HBV-subgenotype is important not only for basic research but also for clinical science and practice as it would help to identify patients at high risk of progressive liver disease or HCC and would allow for more information-based treatment decisions and surveillance strategies.

## Materials and Methods

### Cloning and sequencing of full-length genomes of HBV/D-subgenotypes and introduction of BCP mutations

From archival serum samples of treatment-naïve CHB patients where genotype/subgenotype of infecting HBV was previously established, HBV-DNA was extracted using QIAamp DNA Mini kit (Qiagen, CA, USA). Full-length HBV genome (~3.2 kb) was amplified from the extracted DNA by high-fidelity Taq DNA polymerase (Thermo Fisher Scientific, MA, USA) and primers HBVP1 and HBVP2 (Supplementary Table S1), each bearing unique SapI restriction enzyme site^56^. PCR products were purified by QIAquick gel extraction kit (Qiagen) and cloned separately into pJET1.2/blunt vector with CloneJET PCR cloning kit (Thermo Fisher Scientific). Complete nucleotide sequences of HBV clones were determined using BigDye terminator v3.1 cycle sequencing kit (Applied Biosystems, CA, USA) and different internal primers (Supplementary Table S1) on an automated DNA sequencer (3130, Genetic analyzer, Applied Biosystems). The sequences were compared with representative sequences of ten D-subgenotypes (D1–D10) retrieved from GenBank to reconfirm their subgenotypic affiliations. Additionally, the complete HBx-ORF together with its promoter region (nt. 937–1867) was PCR-amplified with primers HBx_F and HBx_R (Supplementary Table S1) from different D-subgenotype and cloned into Kpn*I*-Hind*III* sites of pGL3*-*basic vector (Promega, WI, USA) and verified by sequencing. The BCP double mutations, 1762T and 1764A were introduced into the wild-type clones of different D-subgenotypes by Site-Directed Mutagenesis (Agilent Technologies) with specific mutagenic oligonucleotides (Supplementary Table S1).

The access to human samples and all experimental protocols were carried out in accordance with the approved guidelines of the Ethical Review Committee of I.P.G.M.E.&R. The written informed consents were obtained from HBV infected patients as well as healthy individuals from whom blood samples were collected.

### Cell culture and transfection

Full-length, linear monomeric HBV of specific D-subgenotype was released from pJET1.2/blunt vector by digestion with 1U of Sap*I*/µg at 37 °C for 12 hours, which was followed by gel purification using QIAquick gel extraction kit (Qiagen). Huh7 cells, seeded into 12-well plates at a concentration of 2 × 10^5^ cells/well, were individually transfected with 1 µg/well of the HBV/D-monomers or pGL3-HBx constructs using Lipofectamine 2000 transfection reagent (Thermo Fisher Scientific) along with pRL-CMV *Renilla Luciferase* Reporter Vector (Promega), which served as transfection normalization control. Six hours post-transfection, culture medium was replaced with fresh Dulbecco’s modified Eagle’s medium (DMEM) (Hi-Media Laboratories Pvt. Ltd., Maharashtra, India) containing 10% fetal bovine serum (FBS) (Thermo Fisher Scientific). The values obtained from each experiment were normalized to Renilla luciferase levels. All experiments were performed in triplicates and repeated at least three times. The data was expressed in fold-change where the expression value of subgenotype-D1 was set at 1.0 and values of other D-subgenotypes were expressed relative to D1.

### Analysis of intracellular HBV-DNA and viral/host gene expression

Seventy-two hours post-transfection, the transfected Huh7 cells were harvested and HBV-DNA was isolated from intracellular core particles^57,58^. Briefly, transfected Huh7 cells were lysed with lysis buffer solution [50 mM Tris-HCL (pH = 8); 1 mM EDTA; 1% NP40] in a total volume of 500 μl. The nuclear pellet was removed by a brief centrifuge at a speed of 10,000 rpm for 2 min at room temperature and the cytoplasmic supernatant was incubated with DNase I (Roche, Basel, Switzerland) and RNase A (Thermo Fisher Scientific) at 37 °C for 3 hours. This nuclease reaction was stopped by incubation at 65 °C for 30 min. The replicated HBV-DNA in the core particles was then isolated by overnight proteinase K (Roche) digestion at 42 °C. The intracellular DNA was extracted with phenol, chloroform, isoamyl alcohol (25:24:1) followed by precipitation with 3 M NaOAc (pH = 5.2) and isopropanol. Finally, the DNA pellet was washed with 70% ethanol, air dried and dissolved in nuclease-free water. The extracted intracellular HBV-DNA was quantified by real-time PCR with SYBR Green Master mix (Applied Biosystems). A 40 cycle real-time PCR was performed in QuantStudio 7 Flex (Applied Biosystems) using primer-pair F5-R4 (Supplementary Table S2) with the following thermal condition: 94 °C for 30 sec, 56 °C for 25 sec, 72 °C for 15 sec. Total RNA was extracted from transfected cells with TRIzol reagent (Thermo Fisher Scientific) and cDNA was generated by reverse transcription with RevertAid Reverse Transcriptase enzyme (Thermo Fisher Scientific). The expression of HBV mRNAs and different host genes, including 18S rRNA, were determined by real-time PCR using specific primer-pairs (Supplementary Tables S2 and S3) and SYBR Green Master mix (Applied Biosystems).

### Assessment of apoptosis

Induction of apoptosis by D-subgenotypes was studied using Annexin-V-FITC/PI kit (BD Pharmingen, CA, USA) according to manuals provided. Briefly, 3 days post-transfection, Huh7 cells were pelleted and resuspended in 1X Annexin-V binding buffer followed by the addition of 5 μL Annexin-V-FITC to the cells and incubation at room temperature in dark for 15 min. Apoptotic cell percentage was measured immediately after addition of 5 μL propidium iodide (PI) using flowcytometry^21^.

### Estimation of cellular reactive oxygen species (ROS)

Generation of ROS in transfected Huh7 cells was measured using cell-permeable DCF-DA (Sigma Aldrich, MO, USA). Briefly, five days post-transfection, cells were harvested and incubated for 30 min in dark at 37 °C in phosphate buffered saline (PBS) containing 5 μM DCF-DA. Cells were then pelleted down by brief centrifugation and dissolved in fresh PBS solution. DCF-DA was converted by ROS to its fluorescent product DCF and the levels of ROS were immediately analysed by measuring mean fluorescence intensity (MFI) of DCF using flowcytometry with an excitation of 485 nm and emission of 530 nm^59^.

### Co-culture of peripheral blood mononuclear cells (PBMCs) of healthy donors and HBV/D-subgenotype-transfected Huh7 cells

PBMCs were isolated from EDTA blood of healthy donors by Histopaque density-gradient centrifugation. Huh7 cells were first seeded at a density of 1 × 10^5^ cells/well in 12-well culture plate and transfected with 1 μg/well of linear monomers of different HBV/D-subgenotypes as mentioned earlier. After 6 hours, all sets of HBV transfected Huh7 cells were mixed with PBMCs (1 × 10^6^ cells/well) and co-cultured for next 3 days. At day 3 of co-culture, the cells were incubated with mitogenic stimulus, Phorbol myristate acetate, PMA (20 ng/ml) and Ionomycin (1 µg/ml) for a period of 2 hours. Following the stimulation, Brefeldin-A (10 µg/ml) was added to the cells. After 3 hours of addition of Brefeldin-A, the PBMCs were taken out gently along with the media, pelleted down by centrifugation, washed with PBS, permeabilized using BD Cytofix/Cytoperm kit (BD Biosciences, CA, USA), stained intracellularly with anti-human TNF-α-APC and IL-6-PE (BD Biosciences) for 20 min at room temperature and frequencies of cells expressing these cytokines were analyzed by flowcytometry.

### Co-culture of LX-2 cells and HBV/D-subgenotype-transfected Huh7 cells

Human hepatic stellate cell (HSC) line, LX-2 was initially maintained in DMEM and 2% FBS for 4–5 passages. Huh7 cells were seeded at a density of 1 × 10^5^ cell/well in lower chamber of 0.4 µm of 24-well transwell plate and transfected with 500 ng of linear monomers of different D-subgenotypes as mentioned earlier. Six hours post-transfection, LX-2 cells were plated on upper chamber of trans-well plates at a density of 1 × 10^4^ cells/well and both cells were then co-cultured in 5% FBS containing DMEM. After 48 hours, total cellular RNA was isolated from LX-2 with Trizol reagent, cDNA was generated and expressions of α-smooth muscle actin (α-SMA) and type-I collagen (Col-I) were determined by real-time PCR using specific primers (Supplementary Table S3). Gene expression was normalized with endogenous 18S ribosomal RNA value.

### MTT assay

Induction of cellular proliferation by different HBV/D-subgenotypes was measured by MTT assay. Huh7 cells, transfected with linear monomers of different HBV/D-subgenotypes were distributed in 96 well plate at a density of 1 × 10^4^ cells/well. After 3 days, the cells were treated with 40 µg/well MTT solution (Sigma Aldrich) and incubated for 4 hrs at 37 °C in dark. The culture medium was then discarded and the cells were suspended with 100 µl of dimethyl sulfoxide (DMSO; Sigma Aldrich) solution. Optical density of the solution was measured on an ELISA plate reader with a test wavelength of 570 nm and a reference wavelength of 630 nm.

### Cell migration assay

Migratory behaviour of Huh7 cells in presence of different HBV/D-subgenotypes was evaluated by wound healing assay. Huh7 cells were seeded into 12-well plates to 80% confluence prior to transfection with different HBV/D-subgenotypes. Twenty-four hours post-transfection, a scratching wound was made by scraping the middle of the cell monolayer with sterile micropipette tip. After washing away all detached cells with PBS, the cells were cultured with DMEM containing 10% FBS for another 48 hours. Images of the wounds were captured soon after scratching and at 48 hour by an inverted microscope, analysed using ImageJ (1.47 v) software and width of the wound over time was calculated to quantify migration rate of the cells^60^.

### Statistical analysis

Data were expressed as mean ± standard deviation (SD). Statistical analysis was performed using GraphPad Prism version 5.0. Statistical comparisons were made using two-tailed paired Student’s t-test, with *p* < 0.05 being considered significant.

## Electronic supplementary material

Supplementary Information
