# Supplementary material for: Distinctiveness in virological features and pathogenic potentials of subgenotypes D1, D2, D3 and D5 of Hepatitis B virus
Source: Sci Rep. 2018 May 23;8:8055. doi: 10.1038/s41598-018-26414-4 (PMC5966457; doi:10.1038/s41598-018-26414-4)
Supplement: Supplementary file 1 — Supplementary Information [file 41598_2018_26414_MOESM1_ESM.pdf]

Supporting Information

Distinctiveness in virological features and pathogenic potentials of subgenotypes D1, D2, D3 and D5 of Hepatitis B virus

Mousumi Khatun[1], Rajiv Kumar Mondal[1], Sourina Pal[1], Ayana Baidya[1], Debasree Bishnu[1], Priyanka Banerjee[1], Amal Kumar Santra[1], Gopal Krishna Dhali[2], Soma Banerjee[1], Abhijit Chowdhury[3] and Simanti Datta[1]

[1]Centre for Liver Research, School of Digestive and Liver Diseases, Institute of Post Graduate Medical Education and Research, Kolkata, INDIA

[2]Department of Gastroenterology, School of Digestive & Liver Diseases, Institute of Post Graduate Medical Education and Research, Kolkata, INDIA

[3]Department of Hepatology, School of Digestive & Liver Diseases, Institute of Post Graduate Medical Education and Research, Kolkata, INDIA

* Corresponding Author: Simanti Datta, Ph.D.

Centre for Liver Research,

School of Digestive and Liver Diseases,

Institute of Post Graduate Medical Education and Research,

244 A.J.C. Bose Road,

Kolkata-700020, INDIA.

Tel- (91)-(033)-22235435; Fax- (91)-(033)-22236383

E-mail- seemdatt@gmail.com

Supplementary table S1: List of primers used for amplification, sequencing and site-directed mutagenesis of HBV

**Primer Name**	**Primer Sequences**	**Location (nt)***
**HBVP1**[a,#] (Sense)	5′-CCGGAAAGCTTGAGCTCTTCTTTTTCACCTCTGCCTAATCA-3′	1821-1841
**HBVP2**[a,#](Antisense)	5′-CCGGAAAGCTTGAGCTCTTCAAAAAGTTGCATGGTGCTGG-3′	1806-1825
**HBx_F**[b] (Sense)	5'-TACTGGTACCAAAGAATGTTTTAGAAAAC -3'	937- 955
**HBx_R**[b] (Antisense)	5'-TGTAAAGCTTTTGAACAGTAGGACATGAAC -3'	1848- 1867
**F10**[c] (Sense)	5' -GACCACCAAATGCCCCTATC -3'	2297- 2317
**F3**[c] (Sense)	5'-CGCCTCATTTTGTGGGTCAC-3'	2801-2820
**F4**[c] (Sense)	5'-CTCAGGCCATGCAGTGGAA- 3'	3164- 3182
**CF**[c] (Sense)	5'-ACTGTTCAAGCCTCCAAGCT-3'	1861-1880
**R2**[c] (Antisense)	5'-AAATTACCACCCACCCAGG-3'	2109-2127
**R3**[c] (Antisense)	5'-AACTGGAGCCACCAGCAG- 3'	57- 74
**R5**[c] (Antisense)	5'-AAAGCCCAAAAGACCCACAAT-3'	997- 1017
**R9**[c] (Antisense)	5´-TAGGAGTTCCGCAGTATGGA- 3´	1265- 1284
**R10**[c] (Antisense)	5'-CAGCCTCCTAGTACAAAGAC-3'	1761-1780
**F7**[c] (Sense)	5'-TGTGCACTTCGCTTCACCTC-3'	1578-1597
**D1-BCP_F**[d] (Sense)	5'- GAGGAGATTAGATTAATGATCTTTGTACTAGGAGGC -3'	1746-1781
**D1-BCP_R**[d] (Antisense)	5'- GCCTCCTAGTACAAAGATCATTAATCTAATCTCCTC -3'	1746-1781
**D2-BCP_F**[d] (Sense)	5'- GAGGAGCTTAGATTAATGATCTTTGTACTAGGAGGC -3'	1746-1781
**D2-BCP_R**[d] (Antisense)	5'- GCCTCCTAGTACAAAGATCATTAATCTAAGCTCCTC -3'	1746-1781
**D3-BCP_F**[d] (Sense)	5'- GAGGAGATTAGGTTAATGATCTTTGTACTAGGAGGC -3'	1746-1781
**D3-BCP_R**[d] (Antisense)	5'- GCCTCCTAGTACAAAGATCATTAACCTAATCTCCTC -3'	1746-1781
**D5-BCP_F**[d] (Sense)	5'- GAGGAGATTAGGTTAATGATCTTTGTATTAGGAGGC -3'	1746-1781
**D5-BCP_R**[d] (Antisense)	5'- GCCTCCTAATACAAAGATCATTAACCTAATCTCCTC -3'	1746-1781

* = nt. positions are given according to HBV sequence with accession no. AF121242 obtained from GenBank. [a] Primers used for full genome amplification of HBV subgenotypes D1, D2, D3 and D5, [#] Günther S, et al. J Virol 1995;69:5437-44. [b] Primers used for amplification of the complete HBx-ORF together with its promoter region (nt. 937–1867) from different HBV/D subgenotypes, [c] Primers used for sequencing, [d] Primers used in Site-directed Mutagenesis

Supplementary table S2: List of primers used for quantification of intracellular HBV-DNA and viral mRNAs by real-time PCR

**Primer Name**	**Primer Sequences**	**Location (nt)***
**F5**[a] (Sense)	5’-GATGTGTCTGCGGCGTTTTA- 3’	376- 395
**R4**[a] (Antisense)	5’-AGAGGACAAACGGGCAACA-3’	462-480
**PreG(N)_F**[b] (Sense)	5'-GACCTCTGCCTAATCATC-3'	1826 - 1843
**PreG(N)_R**[b] (Antisense)	5'-GCTCCAAATTCTTTATAAGG-3'	1913- 1932
**PreC_F**[c] (Sense)	5'- GGTCTGCGCACCAGCACC -3'	1796-1813
**PreC_R**[c] (Antisense)	5'-GCTCCAAATTCTTTATAAGG-3'	1913- 1932
**PreS1(N)_F**[d] (Sense)	5'-TTCCACCAGCAATCCTCTGG-3'	2862-2881
**PreS1(N)_R**[d] (Antisense)	5'-TTGTTGGGATTGAAGTCCC-3'	2942-2960
**PreS2_F**[e] (Sense)	5'-TCCTCAGGCCATGCAGTGG-3'	3162- 3180
**PreS2_R**[e] (Antisense)	5'-AGCCACCAGCAGGGAAATAC-3'	49- 68
**F1**[f] (Sense)	5′-CACAAGAGGACTCTTGGACT-3′	1653-1672
**R10**[f] (Antisense)	5’-CAGCCTCCTAGTACAAAGAC-3’	1761-1780

* = nt. positions are given according to HBV sequence with accession no. AF121242 obtained from GenBank. [a] Primers used for intracellular HBV-DNA quantification by real-time PCR. [b]Primers used for pregenomic-RNA quantification by real-time PCR, [c] Primers used for preC mRNA quantification by real-time PCR, [d] Primers used for preS1 mRNA quantification by real-time PCR, [e] Primers used for PreS2 mRNA quantification by real-time PCR, [f] Primers used for HBx mRNA quantification by real-time PCR

Supplementary table S3: List of primers used for real-time PCR to evaluate the expression of different host genes associated with Apoptosis, Inflammation, Fibrosis and Tumorigenesis pathways

**Primer Name**	**Primer Sequences**	**Amplicon size (bp)**
**Caspase-3_F** (Sense)	5’- AAATGGATTATCCTGAGATGGG-3’	121
**Caspase-3_R** (Antisense)	5’-AATGTTTCCCTGAGGTTTGCTG-3’	
**Bax_F** (Sense)	5′-TGCACCAAGGTGCCGGAAC-3′	94
**Bax_R** (Antisense)	5′-ACCCTGGTCTTGGATCCAGC-3′	
**BcL2_F** (Sense)	5′-GTGGCCTTCTTTGAGTTCGG-3′	109
**BcL2_R** (Antisense)	5′-GCCGGTTCAGGTACTCAGTC-3′	
**TNFR1_F** (Sense)	5'- GGTGACTGTCCCAACTTTGC -3’	164
**TNFR1_R** (Antisense)	5'- GGGTCATCAGTGTCTAGGC -3'	
**Fas_F** (Sense)	5’- TTGTGTGATGAAGGACATGGC -3’	144
**Fas_R** (Antisense)	5’- TTCCATGTTCACATTTGGTGC -3’	
**TRAIL-R1(N)_F** (Sense)	5’- GACCGGCAGAAGCTGAAGGG-3’	136
**TRAIL-R1(N)_R** (Antisense)	5’- ATGAGCTGGTCCCAGGAGTC-3’	
**Grp78_F** (Sense)	5’-CGACCTGGGGACCACCTACT-3’	162
**Grp78_R** (Antisense)	5’-TTGGAGGTGAGCTGGTTCTT-3’	
**ATF4_F** (Sense)	5'-GGGACAGATTGGATGTTGGAGA-3'	63
**ATF4_R** (Antisense)	5'-ACCCAACAGGGCATCCAAGT-3’	
**CHOP_F** (Sense)	5'-CAGAGCTGGAACCTGAGGAG-3'	81
**CHOP_R** (Antisense)	5'-TGGATCAGTCTGGAAAAGCA-3'	
**MCP1(N)_F** (Sense)	5’- GAGACTAACCCAGAAACATCC -3’	144
**MCP1(N)_R** (Antisense)	5’- ATTGATTGCATCTGGCTGAGC - 3’	
**IP10_F** (Sense)	5’- CCAAATCAGCTGCTACTACTCC -3’	138
**IP10_R** (Antisense)	5’- AAGCAGGGTCAGAACATCCAC -3’	

**MIP1b(N)_F** (Sense)	5’- TCTCCAGCGCTCTCAGCACC -3’	171
**MIP1b(N)_R** (Antisense)	5’- AGCACAGACTTGCTTGCTTC -3’	
**CTGF_F** (Sense)	5'- CAGCCCTGACTGCCCCTTCC - 3'	120
**CTGF_R** (Antisense)	5'- AGTCGGTAAGCCGCGAGGGC -3'	
**α-SMA_F** (Sense)	5'-CTGTTCCAGCCATCCTTCAT-3'	70
**α-SMA_R** (Antisense)	5'-TCATGATGCTGTTGTAGGTGGT-3'	
**Col-I(N)_F** (Sense)	5’- TCAAAGTCTTCTGCAACATGG -3’	129
**Col-I(N)_R** (Antisense)	5’- ATGCTCTCGCCGAACCAGAC -3’	
**Ncadherin_F** (Sense)	5’-CACTGCTCAGGACCCAGATCGAT-3’	182
**Ncadherin_R** (Antisense)	5’-TGTCAGAAGCAAGGAAAGTAGC-3’	
**E-Cadherin_F** (Sense)	5’- CTGAAGGTGACAGAGCCTCTG- 3’	124
**E-Cadherin_R** (Antisense)	5’- GATCGGTTACCGTGATCAAAATC- 3’	
**Vimentin_F** (Sense)	5'-GACAATGCGTCTCTGGCACGTC-3'	151
**Vimentin_R** (Antisense)	5'-AAACATCCACATCGATTTGGAC-3'	
**18s_F#** (Sense)	5′-GTAACCCGTTGAACCCCATT-3′	150
**18s_R#** (Antisense)	5′-CCATCCAATCGGTAGTAGCG-3′	
**Rluc_F$** (Sense)	5′-GGAATTATAATGCTTATCTACGTGC-3′	153
**Rluc_R$** (Antisense)	5′-CTTGCGAAAAATGAAGACCTTTTAC-3′	

Primers used for measurement of mRNA levels of 18s rRNA# and *Renilla luciferase* $ by real-time PCR

Supplementary Figure S1

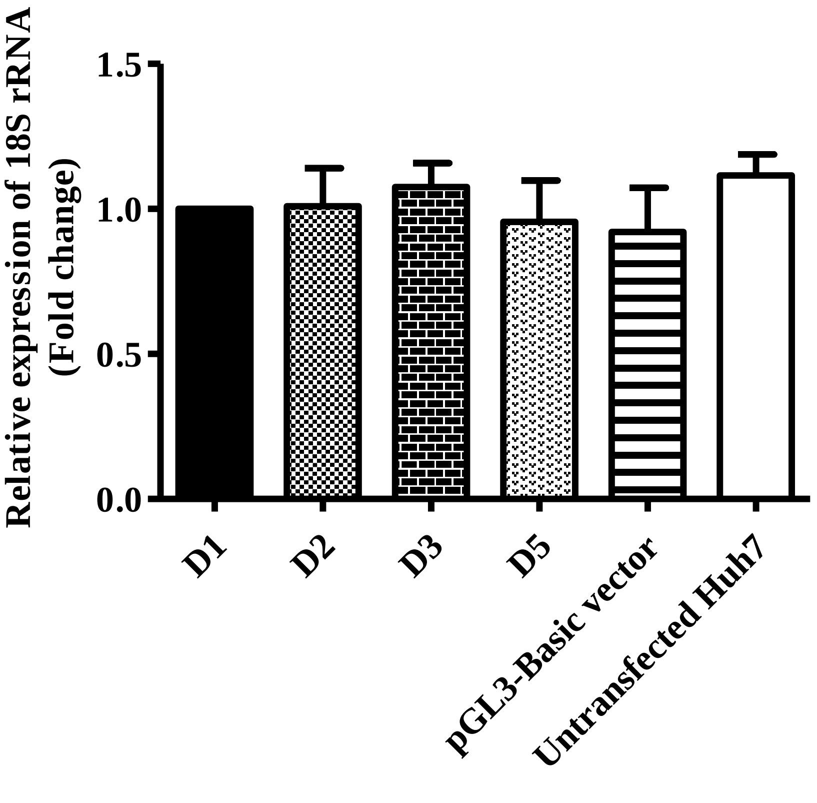

Supplementary Figure S1: Relative expression of 18S rRNA as measured by real-time PCR in Huh7 cells transfected with HBV/D subgenotype D1, D2, D3 or D5 and pGL3-Basic vector DNA (control) as well as in untransfected Huh7 cells.

Supplementary Figure S2

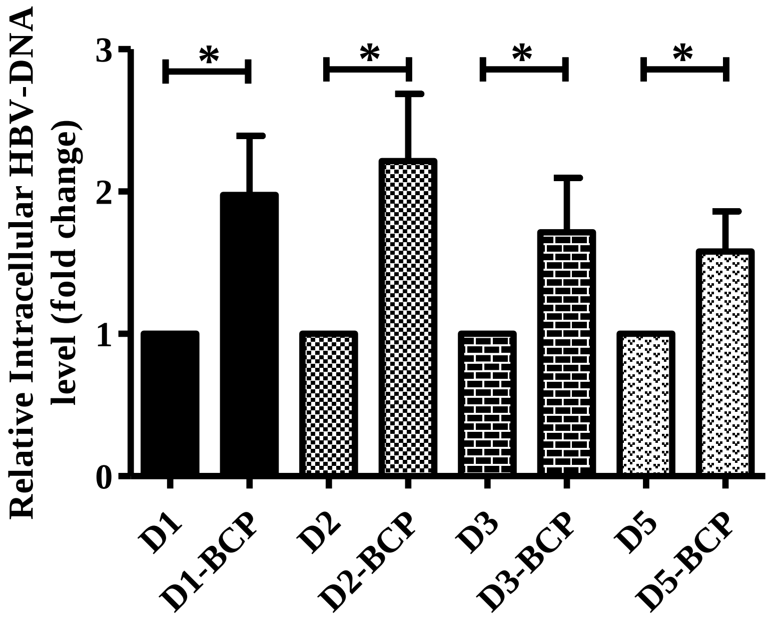

Supplementary Figure S2: Expression of intracellular core-associated HBV-DNA measured by real-time PCR following the transfection of full-length linear monomers of wildtype HBV/D-subgenotypes D1, D2, D3, D5 and their corresponding clones named as D1-BCP, D2-BCP, D3-BCP and D5-BCP respectively each containing basal core promoter (BCP) A1762T/G1764A double mutation into Huh7 cells.
